# Effects of zinc supplementation on subscales of anorexia in children: A randomized controlled trial

**DOI:** 10.12669/pjms.306.6377

**Published:** 2014

**Authors:** Majid Khademian, Neda Farhangpajouh, Armindokht Shahsanaee, Maryam Bahreynian, Mehran Mirshamsi, Roya Kelishadi

**Affiliations:** 1 Majid Khademian, Assistant Professor of Pediatrics, Pediatrics Department, Faculty of Medicine, Child Growth and Development Research Center, Isfahan University of Medical Sciences, Isfahan, Iran.; 2 Neda Farhangpajouh, Resident of Pediatrics, Pediatrics Department, Faculty of Medicine, Isfahan University of Medical Sciences, Isfahan, Iran.; 3 Armindokht Shahsanai, Specialist in Community Medicine, Pediatrics Department, Child Growth and Development Research Center, Isfahan University of Medical Sciences, Isfahan, Iran.; 4 Maryam Bahreynian, MSc (Nutrition), Nutrition Department, Child Growth and Development Research Center, Isfahan University of Medical Sciences, Isfahan, Iran.; 5 Mehran Mirshamsi, MD, Pediatrics Department, Child Growth and Development Research Center, Isfahan University of Medical Sciences, Isfahan, Iran.; 6 Roya Kelishadi, Professor of Pediatrics, Pediatrics Department, Faculty of Medicine, Child Growth and Development Research Center, Isfahan University of Medical Sciences, Isfahan, Iran.

**Keywords:** Anorexia, Children, Randomized controlled trial, Zinc

## Abstract

***Objectives: ***This study aims to assess the effects of zinc supplementation on improving the appetite and its subscales in children.

***Methods:*** This study was conducted in 2013 in Isfahan, Iran. It had two phases. At the first step, after validation of the Child Eating Behaviour Questionaire (CEBQ), it was completed for 300 preschool children, who were randomly selected. The second phase was conducted as a randomized controlled trial. Eighty of these children were randomly selected, and were randomly assigned to two groups of equal number receiving zinc (10 mg/day) or placebo for 12 weeks.

***Results: ***Overall 77 children completed the trial (39 in the case and 3 in the control group).The results showed that zinc supplement can improve calorie intake in children by affecting some CEBQ subscales like Emotional over Eating and Food Responsible.

***Conclusion:*** Zinc supplementation had positive impact in promoting the calorie intake and some subscales of anorexia.

## INTRODUCTION

Macro and micronutrient deficiencies can cause delay in growth and development of children.^1^^,^^2^ Growth delay has many adverse effects, like problems in cognition and immunity.^3^Nutritional insufficiency has different etiologies including socio-economic factors,Lack of enough knowledge about nutritional needs in children is an important reason for Malnutrition^4^ and many families without sufficient information are concerned about the decreased appetite of their children.

Imbalance or monotonous regimen and deficit of micronutrients can play an important role in taking insufficient food ingredients. Some selected micronutrients are shown to have the main roles in this regard like Iron, Zinc and magnesium.^5^^,^^6^ Eating disorders are more widespread between the children than adults and their needs to different micronutrients is dramatically high. Anorexia in children is more important among preschool children who have the most energetic needs and that’s why our study is about preschool children.

Although loss of appetite in some children has behavioral etiologies^7^^,^^8^ but in the others it may be due to diverse factors like micronutrient deficiency, anemia and infections.^1^^,^^5^ In low- and middle-income countries micronutrient deficiencies particularly iron and zinc are prevalent.^7^ There are many reports all over the world about the effects of giving supplements on appetite and growth velocity.^4^^,^^8^^-^^10^ These studies showed controversial results.^4^^,^^9^

Zinc insufficiency in humans is demonstrated for the first time in 1960 in Iran and then some cases were reported from Egypt and Turkey.^11^Zinc has shown to be useful for improving appetite and food intake, through the ghrelin in pigs which is a peptide that secrets from stomach.^11^^-^^13^ In a randomized, double-blind, placebo-controlled, community-based trial,conductedin Peruvian Children with Mild-to-Moderate Stuntingthey concluded that daily provision of 3 mg of supplemental zinc did not affect energy intake.^14^ Moreover, zinc can impact on reduction of anxiety and stress^15^^,^^16^ and this may be another mechanism for the effect of zinc on appetite. It is believed that zinc may be necessary for some cases of anorexia and growth retardation.^9^^,^^13^^,^^16^This study aims to assess the effects of zinc supplementation on improving the appetite and its subscales in children. To our knowledge this is the first clinical trial about the effect of zinc on anorexia subscales in Iran.

## METHODS

This study was conducted from March to December 2013 in Isfahan, Iran. It had two phases.At the first step, after validation of the Child Eating Behaviour Questionaire (CEBQ), it was completed for 300 preschool children aged 2-6 years, who were randomly selected. The second phase was conducted as a randomized controlled trial. Ninety-six of these children were randomly selected, and were randomly assigned to two groups of equal number receiving zinc (10 mg/day) or placebo for 12 weeks. The mothers interviewed to fill in some questionnaires; Based on previous reports an international questionnaire; CEBQ, a 5-point scale, ranging from “never” to “always”, was used after validation in Persian.We validated the questionnaire after writing a letter to the editor, forward and backward translation (all under the supervision of some specialists). This scale is suitable for preschool children and consists of 8 subscales, that are FR (Food Responsiveness), EOE (Emotional Over Eating), EF (Enjoyment Of Food), DD (Desire to Drink), SR (Satiety Responsiveness), SE (Slowness in Eating), EUE (Emotional under Eating), FF(Food Fussiness).

Another questionnaire was designed to examine the general demographic characteristics. Then physical exam and complete labs including cell blood count, urinalysis, urine culture, and stool examwere done for each patient to rule out the organic reasons of anorexia.

The second part was done as a triple-blind study. This trial was approved by ethical committee of Isfahan University of Medical Sciences. It was registered in the Iranian Registry of Clinical Trials, which is a Primary Registry in the World Health Organization Registry Network (Trial registry code: IRCT201302061434N7). Parental written informed consent was obtained prior the study. The main findings would be changes in children food behavior, frequency of iron, zinc, or magnesium deficiencies, intake of calories, protein, carbohydrate and fat. The sample size was calculated as 40 in each group based on Zα=1.96 and Zβ=0.84. Overall,40 cases and 40 controls selected randomly.

***Inclusion criteria: ***Being aged 2 to 6 years; chief complaint of anorexia; not having any disability; not using any supplementation, not having major organic causes of anorexia, i.e. anemia, i.e. hemoglobin under 11g/dL,urinary tract infection, parasitic infection.

***Exclusion criteria: ***Low compliance of family, i.e. non willing of parents to follow the trial, dislike of children to use drug or placebo; gastrointestinal complications in the group receiving zinc.Based on previous studies children were supplemented with zinc (10 mg/day) and placebo for three months.^9^ The supplement and placebo were indistinguishable in colour and form. The cases were followed using syrups monthly. The mothers of the subjects were asked to record three days of food intake for their pre-school children during week days. 

A computer aided nutritional program (Modified NUT4 version 1) by trained nutritionist for the nutritional interview and for the analysis of dietary nutrient intakes. The average daily nutrient intakes, with and without zinc supplement, were compared.

For statistical analysis, we considered intention to treat analysis. Independent t test and Chi square were used by SPSS version 20.0.The statistical significance level was set at P<0.05.

## RESULTS

Overall, 77 children completed the trial (39 in the case and 38.in the control group). Mean level of calories, macro and micronutrients intake in study participants is presented in Table-I.

**Fig.1:**
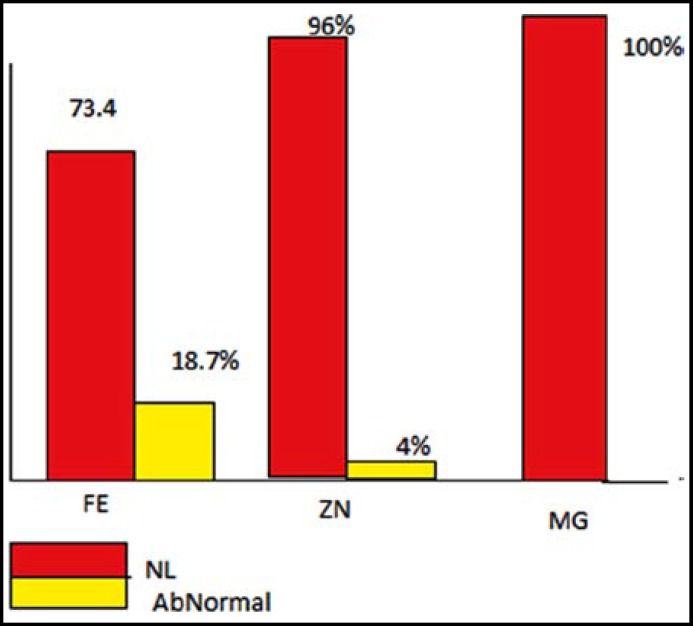
Prevalence of micronutrients deficiency in children according to serum levels of micronutrients

No difference existed in the weight and height and body mass index (BMI) before and after the study (p-value = 0.53, 0.64, 0.4). The prevalence of micronutrients deficiency in children according to serum levels of micronutrients is presented in Figure-1. Comparison of total calorie intake, macro- and micronutrients between two groups is presented in Table-II.

The amount of zinc and magnesium and iron was not significantly different between groups. But the level of zinc increased in both groups. The mean level of serum zinc and magnesium ( mg )was significantly different in the cases and controls(p-value=0.04 and p<0.0001) before and after the study. But the mean of serum iron was the similar in the two groups.

The results showed that zinc supplement could improve calorie intake in children by affecting some CEBQ subscales like Emotional over Eating and Food Responsible (Table-III).

There was no correlation between CEBQ scores and the time spent by mothers to feed their children, and also there was nocorrelation between CEBQ scores and the feeding which is doing being done by mother or anyone else. (p-value=0.2, 0.6, 0.7,respectively).

There was no correlation between father’s education level degree and CEBQ score (0.4)but mother’s education level was significantly associated with the CEBQ score(P=0.002).

## DISCUSSION

Anorexia is one of the most usual chief compliant of children who are referred to the pediatricians. There is controversy that zinc as supplement can improve appétit and different reports exist from different parts of the world.^4^^,^^9^^,^^17^ In Iran no survey has been done in this field. Zinc is an essential trace mineral, so we get it through the foods we eat.Zinc is the most common mineral in the body and is found in every cell, next to iron.^18^

This study shows that zinc supplement can improve calorie intake in children; (Table-II) but in a survey on children up to 2 years old in USA in 2008 there were no differences in dietary energy intake after intervention by zinc supplement.^17^ Also, in a study done about the effect of zinc on fat-free mass on peruvian children, those who received the zinc supplement had a greater increase in fat free mass but there were no differences about prevalence of repoted poor appetite between case and control group after receiving zinc supplement.^14^ Good appetite is a useful predictor of excessive weight gain.^19^ The observed increase in appetite may be due to the growth of children and because when they get bigger or older their calorie intake may also increase, but at the present study the differences between two case and control groups (p-value=0.00) even without significant rise in serum level.

In our 3 months follow up, no difference existed in weight and height and BMI before and after study (p-value=0.53, 0.64, 0.4) but if longer follow up was done, significant differences might be found.

It is suggested that using a continuous visual analogue scale of the appetite item would have provided greater sensitivity compared to the 5 point scales used here but today the best scaling in preschool children appetite is CEBQ, that was developed for young children. The subscales in the CEBQ found to have independent predictive power in our study were food responsiveness, emotional over eating and emotional under eating. These variability in CEBQ subscales has also shown to be partly genetic in origin.^19^

In our study, some differences existed between some subtypes of CEBQ scores in two groups as shown in Table-III. Emotional over eating (EoE) ,which is a part of food approach subscale on the CEBQ questionnaire, was statistically different in the case group, but not in control group. So it may lead to take more calories in case group. We showed that in control group, EUE subscale was different before and after the study, but it had no effect on calorie intake and this result was like the other studies.^20^

The serum concentration of zinc is the most commonly used indicator of zinc status.^5^ Although it is influenced by variety of factors such as infection, stress, pregnancy and growth velocity, which limit its diagnostic value. However as mentioned earlier, about 4% of children had serum zinc concentration below the normal level ,but this prevalence was 44% in a study in Ethiopia and 25% in a study in Mexican children. The main nutritional deficiency in this study was iron deficiency with a 18.7% prevalence.

**Table1:** Mean level of calories, macro and micronutrient intake in study participants

**Variables (unit)**	**Mean (SD)**
Total calorie intake(Kcal)	956 (22.2)
Carbohydrates (g)	158 (44.1)
Protein (g)	82 (13.9)
Fat(g)	19 (10.6)
Zinc (u)	0.3 (0.2)

**Table 2:** Comparison of total calorie intake, macro- and micronutrients between two groups

**Variables**	**Before placebo** **Mean (SD)**	**After placebo** **Mean (SD)**	**Before zinc** **Mean (SD)**	**After zinc** **Mean (SD)**	**p-value**
Total calorie intake	960.5 (22.2)	953 (22.4)	930 (136)	1022 (160)	0.00
Carbohydrate	152 (44.1)	148 (42.1)	155 (30)	172 (35)	0.00
Protein	45 (13.9)	47.4 (17.2)	40.6 (14)	43.6 (12)	0.00
Fat	18.4 (10.6)	10.6 (10.4)	16 (7)	17.8 (7.8)	0.00
Zinc	0.36 (0.2)	0.38 (0.22)	0.36 (0.2)	0.38 (0.2)	0.00

**Table3:** Comparison of anorexia indicators due to CEBQ questionnaire between two groups

**Groups**	**Subtypes**	**p-value**
Control	CEBQ1-CEBQ2	0.99
	FR1-FR2	0. 3
	EOE1-EOE2	0.09
	DD1-DD2	0.3
	SR1-SR2	0.07
	SE1-SE2	0.6
	EUE1-EUE2	0.011*
	FF1-FF2	0.6
	EF1-EF2	0.11
Case	CEBQ1-CEBQ2	0.85
	FR1-FR2	0.03*
	EOE1-EOE2	0.006*
	DD1-DD2	0.16
	SR1-SR2	0.26
	SE1-SE2	0.243
	EUE1-EUE2	0.08
	FF1-FF2	0.42
	EF1-EF2	0.07

FR = Food Responsiveness, EOE = Emotional Over Eating, EF = Enjoyment of Food,

DD = Desire to Drink, SR = Satiety Responsiveness, SE = Slowness in Eating,

EUE = Emotional Under Eating, FF = Food Fussiness.

* : P<0.05,

Actually, weight disorders are an important global problem with several adverse health effects,^21^^-^^24^and some microelements can have beneficial roles in this regard.^25^^-^^27^ Prevention and control of micronutrient deficiency might have many beneficial long-term health impacts.

*Study limitations and strengths:* The main limitation of this study was its short duration of follow up. With longer follow up, some of current borderline results might become significant. The strength of the study is its novelty in studying the subscales of anorexia by validated questionnaire.

## CONCLUSION

Zinc can improve appetite of 2-6 years old children by affecting some CEBQ subscales like EOE and FR .Though zinc deficiency in our study was estimated to have a low frequency of about 4% ,and zinc deficiency seems not to be a main problem in Iranian children, but positive impact of this micronutrient showed that it can promote the calorie intake in anorexic children.

## Notes

***Source of funding: ***This study was funded by the Child Growth and Development Research Center, Isfahan University of Medical Sciences
